# Integrated Analysis Identifies Interaction Patterns between Small Molecules and Pathways

**DOI:** 10.1155/2014/931825

**Published:** 2014-07-13

**Authors:** Yan Li, Weiguo Li, Xin Chen, Hong Jiang, Jiatong Sun, Huan Chen, Sali Lv

**Affiliations:** ^1^Department of Bioinformatics, School of Basic Medical Sciences, Nanjing Medical University, Nanjing 210029, China; ^2^Faculty of Health Sciences, University of Macau, Macau

## Abstract

Previous studies have indicated that the downstream proteins in a key pathway can be potential drug targets and that the pathway can play an important role in the action of drugs. So pathways could be considered as targets of small molecules. A link map between small molecules and pathways was constructed using gene expression profile, pathways, and gene expression of cancer cell line intervened by small molecules and then we analysed the topological characteristics of the link map. Three link patterns were identified based on different drug discovery implications for breast, liver, and lung cancer. Furthermore, molecules that significantly targeted the same pathways tended to treat the same diseases. These results can provide a valuable reference for identifying drug candidates and targets in molecularly targeted therapy.

## 1. Introduction

Recently, with the development of molecular biology techniques, molecularly targeted therapy has been applied in clinical practice [1, 2]. In cancer research, molecularly targeted therapy aims to identify the agent for a known therapeutic target. The agent can modify the expression or activity of the target during the growth and progression of the cancer [3]. Unfortunately, the set of drug candidates must be determined by rigorous and repeated experiments [4], a process that is beset with difficulties and is usually time consuming.

Small molecules act by simultaneously participating in multiple biological processes and triggering a variety of changes that lead to diverse reactions. A phenotype is always caused by a series of complex molecular reactions. A pathway embodies complex interactions between small molecules and macromolecules, and most pathways are interrelated [5, 6]. Thus, it is of great biological importance to detect the links between small molecules and their target pathways and to perceive the intervention effects of small molecules on disease through these pathways [7]. In addition, the concept of biochemical pathways aids in understanding the mechanisms of cancer [8]. Considering a pathway as a functional unit facilitates the unravelling of the mode of action for small molecules [9]. A number of studies have reported drug targeted pathway that was the effective therapeutic approach in treating cancer [10–15]. For example, a hedgehog pathway inhibitor, vismodegib, has recently been approved by the US FDA for the treatment of skin cancer, while several drug candidates for the Wnt pathway are entering clinical trials [12]. Azole drugs, which are commonly used in infection treatment, play a part in azole therapy by targeting the sterol biosynthetic pathway [11]. The findings of Chian and his colleagues demonstrated that luteolin inhibits the NRF2 pathway in vivo and can serve as an adjuvant in the chemotherapy of NSCLC [10]. Collins and Workman reported that there were several kinds of potential drug targets: oncogene products downstream, proteins in a key pathway and oncogenic support processes [16]. Disease-related pathways that are affected by the intervention of small molecules are more likely to include target genes [17]. Therefore, employing computational methods to explore the links between small molecules and pathways provides a new perspective for molecularly targeted therapy. With the ongoing research into genome, proteome, and transcriptome, various databases for small molecules and pathways emerge one after another, such as Connectivity Map [18, 19], DrugBank [20, 21], CTD [22], KEGG [23], and the NCBI PubChem [24]. These databases provide abundant data resources for high-throughput analysis, and this availability allows the creation of a computational method to construct the links between small molecules and their target pathways, which can provide complementary and supporting evidence to experimental studies.

Based upon the above considerations, we have proposed a novel method to detect the links between small molecules and pathways. First, differentially expressed genes related to diseases were identified and enriched into KEGG pathways. Next, molecules that target each pathway were identified by Connectivity Map. We further constructed a link map between the molecules and their target pathways and analyzed the topological features of the link map. Moreover, by applying this method to a chosen set of data, we identified three link patterns. We also found that if molecules significantly targeted the same pathways, then they tended to treat the same disease. Besides, we provide potential candidate for drug experiment by mining and predicting medicinal small molecules and their target pathway in in silico method. This application may provide valuable information to molecularly targeted therapy from a pathway-based perspective.

## 2. Materials and Methods

### 2.1. Data Sources

The microarray data were downloaded from Gene Expression Omnibus (http://www.ncbi.nlm.nih.gov/geo/, accession number GSE5364). In this study, we used three datasets including both tumour tissues and adjacent normal tissues (tissue type/tumour/normal: breast/183/13, liver/9/8, and lung/18/12). In addition, another dataset was added into each type of cancer to establish link map as well as to predict target relationship. The data included both tumour tissues and adjacent normal tissues (tissue type/tumour/normal: breast (GSE15852)/43/43, liver (GSE9166)/15/18, and lung (GSE7670)/27/27).

Connectivity Map (http://www.connectivitymap.org/cmap/) consisted of more than 7,000 gene expression profiles treated with 1,309 small molecules. These expression profiles represented about 6,000 instances, each of which comprised a treatment and vehicle pair. By comparing the expression pattern similarity of the input genes and the genes perturbed in Connectivity Map instances, a list of molecules related to the input genes would be identified.

### 2.2. Screening of Differentially Expressed Genes

For each dataset, probes corresponding to more than one gene were discarded. Log_2_ transformation of the expression value was performed for each probe, and the data was normalized by the quantile normalization method. Up- and downregulated probes were determined according to their fold difference [25]. All probes were then mapped to Entrez gene IDs using mean values. Differentially expressed genes were screened by significance analysis of microarrays (SAM) [26] with FDR = 0.001.

### 2.3. Detection of Significant Links between Small Molecules and Pathways

**Figure 1:**
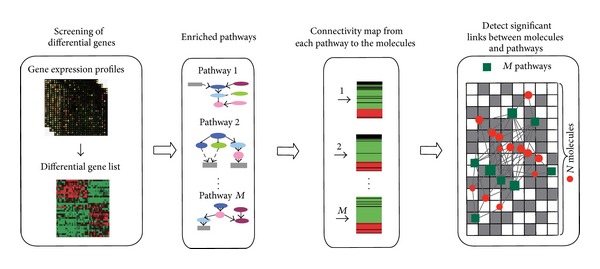
Flow chart for the construction of a link map between molecules and their target pathways. First, the gene expression profiles for each cancer were obtained from both tumours and adjacent normal tissues and differentially expressed genes were screened. Second, enriched pathways were obtained by enriching differentially expressed genes into KEGG pathways. Third, differential genes in each enriched pathway were input into Connectivity Map to identify the small molecules related to each pathway. Finally, link map between small molecules and pathways (the M-P link map) was constructed based on the significance level of their association. In the background grid, the corresponding cell is coloured grey if small molecules significantly link to the pathway in the M-P link map, while the cell was white if such links did not exist. The front network was a visualisation of the background grid in which each pathway node was represented by a green rectangle, while each molecule node was represented by a red circle. The node size increased with the number of first-order neighbours in the link map.

The differentially expressed genes from GSE5364 were annotated into KEGG pathways using WebGestalt [27] (http://bioinfo.vanderbilt.edu/webgestalt/). When it came to data used to predict target relationship, we manipulated DAVID to annotate pathways and carried out corresponding enrichment analysis. Then, statistically enriched pathways, which are potentially relevant to diseases, were obtained using hypergeometric test (*P*≤0.05). Differentially expressed genes were partitioned into enriched KEGG pathways. For each pathway, up- and downregulated probes corresponding to differential genes were input into Connectivity Map. The small molecules that were significantly related to a certain pathway were identified according to *P* value from the permuted results given by Connectivity Map. In this way, small molecules for all the enriched pathways could be found. Thus, we could construct an adjacency matrix between the small molecules and the pathways. The elements of the matrix were set to one when *P* ≤ 0.01; otherwise, they would be set to zero. Suppose the adjacency matrix was *A*. When *A*_*ij*_ = 1, then that small molecule *i* significantly targets pathway *j*. A bipartite M-P network of molecules and pathways can be constructed based on the adjacency matrix. The nodes of the M-P network were small molecules and pathways, respectively. A link was placed between a molecule and a pathway if the molecule significantly targets the pathway. The M-P network was also called the M-P link map.  Figure 1 shows the process of constructing the M-P link map.

### 2.4. Detect the Links Where a Single Molecule Robustly Targets a Single Pathway from the M-P Link Map

Two types of unipartite links were derived from the M-P link map: molecule-molecule links and pathway-pathway links. Such a relationship between two molecules will exist if the two molecules significantly target the same pathways, and such a relationship between two pathways will exist if the two pathways were significantly targeted by the same molecules. The two unipartite links were inferred by the cumulative hypergeometric distribution [28]. The details were as follows.

We suppose that two different molecules target *n*_1_ and *n*_2_ pathways. The size of the intersection and the union of their target pathways were share and *n*, respectively. Then, the significance level of the two molecules targeting the same pathways was calculated as follows:

(1) P=1−∑i=0share−1pi,

where

(2) pi=(n2i)(n−n2n1−i)(nn1), i=0,1,2,…share−1.

Similarly, the significance level of two pathways targeted by the same molecules could be calculated. We further extracted the links where a single molecule robustly targets a single pathway from the original M-P link map using molecule-molecule links and pathway-pathway links. Robust links between molecules and pathways were defined as follows: small molecule A and some other molecules significantly target pathway B; at the same time, pathway B and some other pathways were significantly targeted by molecule A.

## 3. Results

### 3.1. The Topological Characteristics of the M-P Link Map

**Figure 2:**
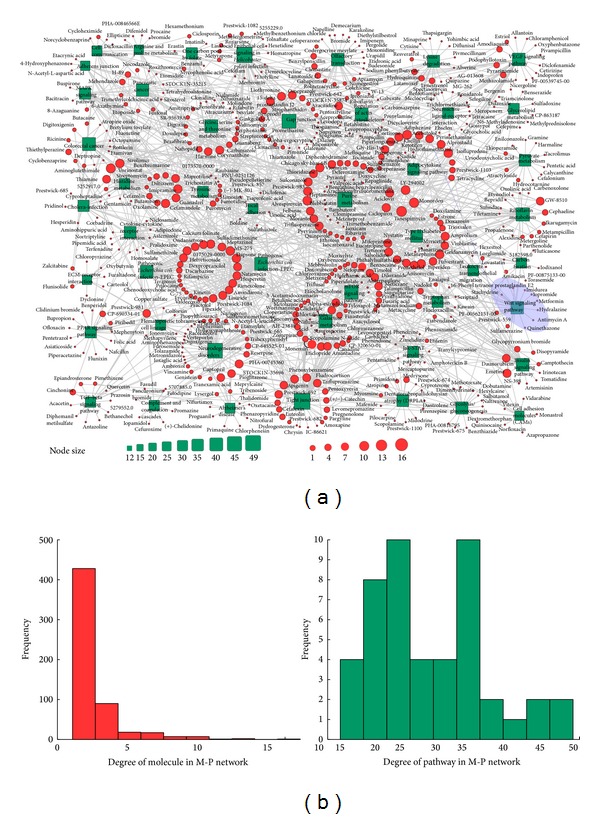
Visualisation of the M-P link map for breast cancer and its degree distribution. (a) The red circles and green rectangles correspond to the small molecules and pathways, respectively. Node size is proportional to the degree of the node. (b) The degree distribution of small molecules and pathways.

We proposed a method to construct a link map between small molecules and pathways (M-P link map), as described in Section 2. The method was applied to analyze breast cancer, liver cancer, and lung cancer, as described in the Materials and Methods. M-P link maps for these three cancers from GSE5364 were constructed. The M-P link map for breast cancer and its degree distribution are shown in Figure 2. The heatmap for M-P link map of breast cancer is shown in Figure 4(a). The M-P link maps and degree distributions for liver and lung cancers are shown in Figures S1 and S2, respectively (see Figures S1 and S2 in Supplementary Material available online at http://dx.doi.org/10.1155/2014/931825). Their corresponding heatmaps are shown in Figures S3(a) and S4(a).

For the M-P link map in Figure 2, the degree of molecules followed a power law distribution. Relatively few molecules were of high degree, targeting multiple pathways. Instead, the majority of small molecules were of low degrees because they target few pathways. Of the 571 small molecules in the M-P link map for breast cancer, eight (1.4%) molecules had degrees larger than ten, while 300 (52.5%) molecules have degree one, meaning that each molecule in the latter category links to only one pathway. Of the 263 small molecules in the M-P link map for liver cancer, only one (0.38%) molecule had the highest degree eight, and 183 (69.6%) molecules had degree one. Of the 276 small molecules in the M-P link map for lung cancer, only one (0.36%) molecule had degree eight, and 196 (71.0%) molecules had degree one.

**Figure 3:**
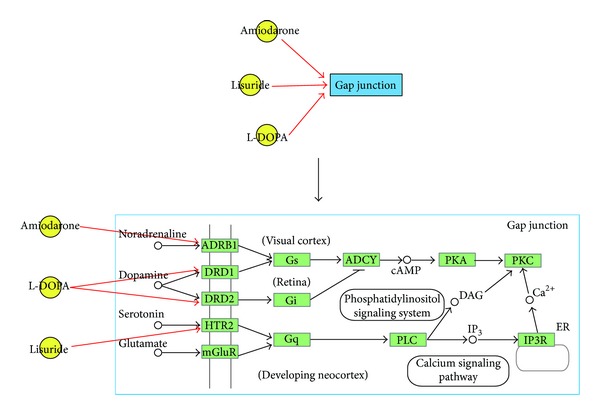
The case of three small molecules amiodarone, L-DOPA, and lisuride targeted gap junction. The yellow points represent small molecules, while the blue box represents gap junction pathway. The green boxes represent the genes in gap junction.

The degree of the pathways did not show a similar distribution to the degree distribution for small molecules. There were no significant differences in the number of pathways over various degrees. The average degree of the pathways was higher than that of the small molecules. Additionally, the degrees of the pathways were all larger than one. This result indicated that one pathway might not be specifically targeted by one small molecule and was consistent with the biological fact that one pathway might be targeted by distinct small molecules through different genes in the pathway.  Figure 3 depicts an instance of gap junction, which turned out to be target pathways of amiodarone, L-DOPA, and lisuride. By directly interfering ADRB1, DRD1, DRD2, and HTR2 of the pathway, amiodarone, L-DOPA, and lisuride established target relationships with gap junction pathway. Here, gap junction partially manifests the target protein-related section rather than the whole pathway.

### 3.2. The Link Patterns in the M-P Link Map

**Figure 4:**
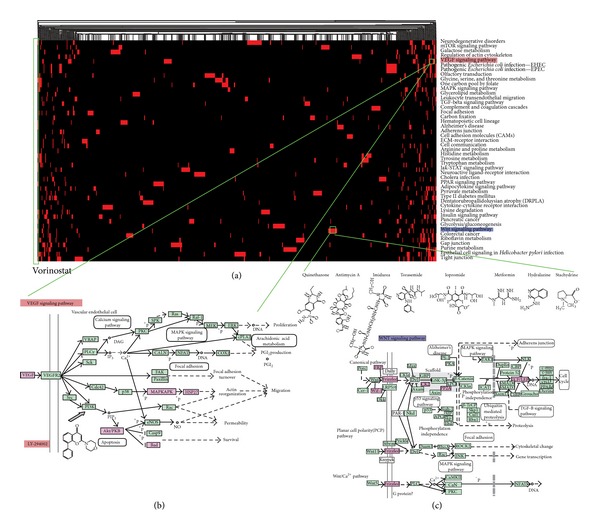
Hierarchical clustering in the M-P link map for breast cancer from GSE5364. (a) Hierarchical clustering between 571 small molecules and 47 metabolic pathways. The corresponding cells are coloured red, where small molecules link to the pathways in the M-P link map. The labels for the corresponding pathways are shown on the right of the figure. (b) Zoomed-in plot of an sM-sP link between LY-294002 and the VEGF signaling pathway. The gene indicated by the arrow is the drug target of LY-294002. Differentially expressed genes in this pathway are coloured pink, while other genes in green are human disease-related genes. (c) Zoomed-in plot of mM-sP links between eight small molecules and the Wnt signaling pathway. These eight small molecules target only the Wnt signaling pathway. The differentially expressed genes in this pathway are coloured pink, while other genes in green are human disease-related genes.

Based on the M-P link map, there are several link patterns that help reveal the intervention features of molecules on their target pathways. The first pattern is that a single molecule targets multiple pathways (sM-mP). Molecules from this pattern can intervene in multiple pathways. Most tumours involve the regulation of multiple genes and processes. There are usually many potential targets in these tumours [29]. All cell signal transductions cannot be blocked by inhibiting a single receptor or target. Therefore, molecules with the sM-mP pattern may be considered as antineoplastic candidates, but they may cause many side effects. For breast cancer from GSE5364, we found that molecules with the sM-mP pattern, which had a high degree, could be used for cancer treatment.  Table 1 provides detailed descriptions for eight small molecules with degrees not less than ten. Among these molecules, LY-294002 is a potent PI3K inhibitor. PI3K is related to human tumourigenesis, including breast cancer [30]. Vorinostat has also been shown to correlate with breast neoplasm. Tanespimycin, monorden, alvespimycin, and monensin are directly related to the occurrence of cancer. For example, vorinostat targets 12 pathways, which are marked with a green box at the left in Figure 4(a). The descriptions of high degree small molecules of the other two sets of data can be found in Tables S1 and S2.

The second link pattern is where a single molecule targets a single pathway robustly (sM-sP), which can be extracted from the original M-P link map using molecule-molecule links and pathway-pathway links (as described in Section 2). Disease-specific links may be identified in sM-sP links. In the case of breast cancer from GSE5364, the link between LY294002 and the VEGF signaling pathway belongs to the sM-sP pattern. Figure 4(b) shows the possible mechanism through which LY294002 intervenes in the VEGF signalling pathway. LY-294002 is a potent PI3K inhibitor. PI3K, a target gene of LY294002, is involved in the VEGF signaling pathway [31]. The VEGF signaling pathway is closely related to angiogenesis, and PI3K is a key regulator of this biological process. PI3K is related to human tumourigenesis, including breast cancer, lung cancer, melanoma, and lymphoma. LY-294002 may first target PI3K, which is located at the upstream of the VEGF signaling pathway, thereby leading to the differential expression of downstream genes. Another example of an sM-sP link is between isoniazid and the glutathione metabolism pathway in the link map for liver cancer from GSE5364 (Figure S3(b)). Isoniazid is an antibacterial agent used primarily as a tuberculostatic. It remains a preferred choice for the treatment of tuberculosis. ABAT, a target of isoniazid, is involved in glutamate metabolism. According to KEGG pathway, glutamate metabolism is part of glutathione metabolism. A third example of an sM-sP link is between alsterpaullone and the insulin signaling pathway in the link map for lung cancer from GSE5364 (Figure S4(b)). Alsterpaullone is usually involved in protein kinase activity [32]. GSK3B, which is one of its target genes, can inhibit tumourigenesis and tumour diffusion. The GSK3B enzyme also plays crucial roles in various tumours, including breast, colon, kidney, and stomach cancers. It has been examined as a potential target in cancer therapy [33]. If the GSK3B enzyme is inactivated, the risk of occurrence of liver cancer will increase [34].

The third link pattern is where multiple molecules target a single pathway (mM-sP). In this pattern, one pathway is targeted by many molecules that do not link to any other pathways. Molecules in this pattern have few side effects. Therefore, these small molecules may be candidate adjuvant drugs for anticancer agents. Additionally, parts of the molecules tend to have similar efficacy. For the first instance, there are a total of 31 small molecules that target the Wnt signaling pathway in breast cancer from GSE5364. There are eight molecules with degree one, which are shaded light slate blue in Figure 2(a). These eight molecules are stachydrine, torasemide, quinethazone, hydralazine, imidurea, antimycin, iopromide, and metformin. The first four are used to treat hypertension and cardiovascular disease. Details of their intervention with genes in the Wnt signaling pathway are shown in Figure 4(c). We may identify target genes for these molecules from differential genes in this pathway. The second example is a set of 15 small molecules targeting only the focal adhesion in liver cancer from GSE5364 (Figure S3(c)). Orciprenaline, budesonide, and hydrocortisone are used to treat asthma or asthma-related diseases. Trihexyphenidyl, tiletamine, and thiocolchicoside have sedative and muscle relaxant effects. The case is shaded light slate blue in Figure S1(a). Another instance is the separate subnetwork shaded by light slate blue in the M-P link map for lung cancer from GSE5364 (Figure S4(c)). The central node is the cell communication; this node is connected to 16 small molecules with degree one, of which 14 have relatively clear efficiency. Six molecules are mainly used as either antibacterial or anti-inflammatory agents, and four molecules are used for the treatment of psychoses. For the molecules in this link pattern, we can explore drug substitution or efficacy prediction for small molecules. Small molecules affecting the same gene ontology (GO) modules could be considered for drug substitution or efficacy prediction [35].

### 3.3. Potential Cocurative Effects of Molecules That Significantly Target Same Pathways

For some diseases, single-agent therapy may not be sufficiently effective [36]. To increase the effectiveness of drugs, combination therapy was used to enhance their efficacy by synergistic effect. Links between small molecules are implied in the M-P link map. We can also construct molecule-molecule links if two small molecules are both used to treat the same disease. We call these small molecule pairs cocurative molecule pairs. Small molecules were input into CTD to query diseases related to them. We calculate the overlap between cocurative molecule pairs and molecule pairs targeting the same pathways. The proportions of overlap in breast cancer, liver cancer, and lung cancer from GSE5364 are 41.3%, 41.2%, and 22.7%, respectively. These overlaps are statistically significant by Fisher's exact test with *P* < 0.001 [28]. The results showed that the small molecules that significantly targeted the same pathways tended to treat the same diseases. This conclusion has been mentioned in a recent published work [37]. Such small molecule pairs may be used as alternative medications or as a drug combination. As our knowledge of small molecules accumulates, the proportion of known pairs will increase. At the same time, the efficacy of molecules can be predicted more easily if the efficacies of corresponding molecules which target the same pathways are known. However, when administered together, antagonism between the cocurative drugs should be considered to reduce adverse reactions.

### 3.4. Common Small Molecules and Pathways Shared by Three Cancers

From the M-P link map, we found that there were 71 small molecules shared by three cancers from GSE5364. All molecules with degrees of five or more from the three cancers were included in this group. Of these 71 molecules, 58 were recorded in existing databases. Nine of these 58 molecules can be directly used as antitumour agents or directly impact tumour-related proteins. They are trichostatin A, geldanamycin, azacitidine, puromycin, streptozotocin, vorinostat, genistein, camptothecin, and sulfadimethoxine. The five common pathways are ECM-receptor interaction, focal adhesion, insulin signalling, cell communication, and Type II diabetes mellitus. The first three pathways are related to cell differentiation, proliferation, and apoptosis. Pathways appearing in only one cancer seldom show these characteristics. In liver cancer, for example, the majority of the pathways are involved in biological processes related to the metabolic functions of the liver. For instance, glycerophospholipid metabolism and glycerolipid metabolism are part of lipid metabolism, while fructose and mannose metabolism and pyruvate metabolism belong to carbohydrate metabolism, and glutathione metabolism metabolises abnormal amino acids. In particular, lipid metabolism and pyruvate metabolism are closely related to liver cancer [38–40].

### 3.5. Analysis and Validation of Link Relationship

**Figure 5:**
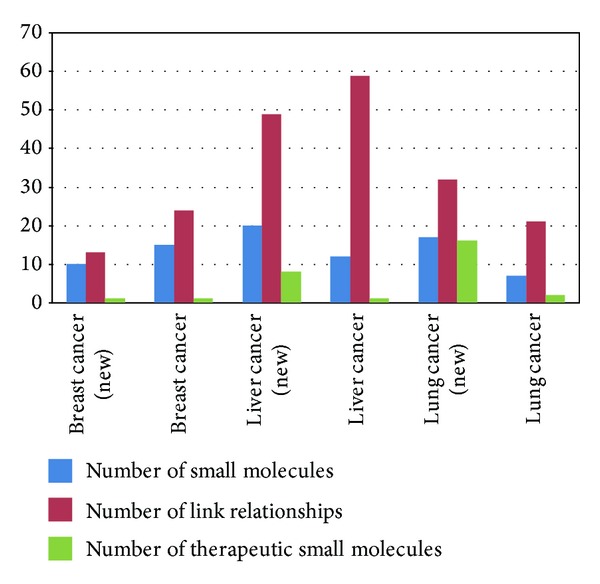
A chart indicates the validated six datasets of three cancers. The blue bars represent the validated small molecules, while the red bars turn out to be the validated M-P link relationships. Besides, the green bars are the small molecules for anticancer drugs proved.

To validate the link relationships between small molecules and pathways, we developed the reliability analysis. We used three sets of data from GEO, that is, GSE15852, GSE9166, and GSE7670, respectively, to construct M-P link. As a consequence, we obtained M-P links corresponding to six sets of data. Actually, there were two sets of data in each type of cancer. Firstly, for the links between 571 small molecules and 47 pathways from our method for breast cancer dataset, we extracted known targeted genes for each small molecule using DrugBank database and identified whether these targeted genes were members of 47 pathways. If so, the predicted target relationship can be validated. In the results, the relationships of 15 small molecules targeting 25 pathways have been identified. As a validated example, genistein was identified by CTD database, which was an antineoplastic and antitumor agent by targeting leukocyte transendothelial migration [41]. Furthermore, we added another breast cancer dataset to confirm our analysis and then identified the relations of 10 small molecules targeting 13 pathways. Cheng et al. found that quercetin induced tumor-selective apoptosis through downregulation of valine, leucine, and isoleucine degradation [42]. The overlap between two breast cancer datasets was 402 small molecules and showed high consistency. We also furthered the validation analysis by comparing the relationship of small molecules targeting pathways to DrugBank and CTD databases for liver and lung cancers and known targeted therapy results were available in Table S3 [43–45]. The validation results were summarised in Figure 5.

## 4. Discussion and Conclusions

In this study, we constructed a link map between small molecules and pathways with Connectivity Map database and analysed the topological properties of this link map. Pathways were regarded as drug targets, and we studied small molecules' effects on disease from the perspective of these pathways, which provides valuable information for molecularly targeted therapy.

To detect pathways that were targeted by small molecules, disease-related differentially expressed genes are first enriched into pathways. In this way, differentially expressed genes were divided into various subsets based on KEGG pathways they participate in. Thus, it is possible to detect potential candidate small molecules from the perspective of the pathways that include differentially expressed genes. Although there is low reproducibility of differentially expressed genes between diverse data sources for the same kind of diseases, the expression correlation of those genes is high [46]. This result indicates that separate experiments can identify different but functionally correlated sets of differential genes. In the proposed method, we selected one dataset for each cancer on the same platform. Nevertheless, it is still possible to identify significant links between small molecules and pathways. By comparing the expression pattern similarity of the differentially expressed genes enriched in a pathway and the genes perturbed in Connectivity Map instances, molecules that intervene in the pathway were identified. Then, we constructed the link map between small molecules and all pathways. From the target pathway of the molecules, we can further screen their target genes.

The proposed approach established the mapping between disease-related pathways and small molecules. After analysing three cancer datasets from GSE5364, we were able to describe specific characteristics of the link maps of small molecules with high degrees in the sM-mP pattern that were always used for cancer treatment. Most tumours involve the regulation of multiple genes and processes, and there are usually many potential targets for treating tumours [29]. Thus, small molecules with high degrees may be considered as antineoplastic candidates. For small molecules in the mM-sP pattern, some of them are of similar efficacy. This similarity facilitates the prediction of drug efficacy and the identification of new efficacies for existing drugs. In the established link map, the number of genes shared by both pathways indeed plays part in impacting the number of their cotargeted small molecules. In spite of this, it is not the determining factor. By application of KS-like test of Connectivity Map to evaluate the expression deregulation caused by small molecules, the function of genes that remarkably interfered with small molecules became increasingly obvious. Even though the two pathways might share the identical genes, their function can vary from pathway to pathway, which would influence the number of corresponding small molecules that had been mined.

Although there are common molecules and pathways for the three cancers, there is no identifiable overlap among the M-P links in our datasets. This result is not surprising. In the process of pathway enrichment analysis for the three datasets, diverse differentially expressed genes may be enriched into different subpathways in the same pathway. When the genes are input into Connectivity Map to detect significant molecules, different genes in the same pathways may link to distinct molecules. Thus, in different datasets, the same pathway may be targeted by different molecules. Thus, there may not be any overlap among the M-P links for the three cancers.

Meanwhile, in order to validate the stability of the results, we added another set of data in each type of cancer to construct link maps. By comparing to the original data, we found a good repeatability. The number of repeat small molecules between two sets of breast cancer data is 402; when it comes to lung cancer and liver cancer, the numbers are 218 and 276, respectively. Axon guidance, which proved to be small molecules of tumor treatment in the previous analysis, was found in both sets of lung cancer data. In breast cancer data, we had found the meticrane targeting PPAR signaling pathway that had been reported before. Meanwhile, we found the known clomipramine targeting metabolism of xenobiotics by cytochrome P450 pathway in liver cancer.

In the future, we can expand the candidate target pathways affected by small molecules through the integration of multiple cancer datasets. As the information on pathways and small molecules accumulates, more comprehensive results will be obtained. In our future work, we will expand the types of cancer and provide creative thinking for molecularly targeted therapy from the perspective of systematic biology. We will focus our creative thinking on further researching mechanisms of drug functions of validated small molecules and make a contribution to the development of clinical anticancer drugs. Besides, we are taking other types of targets of small molecules, namely, miRNA, to specify the target relationships between small molecules and pathways. We will explore the molecule-impacted target pathway from different perspectives.

## Supplementary Material

Figure S1: Visualisation of the M-P link map for liver cancer from GSE5364 and its degree distribution.
Figure S2: Visualisation of the M-P link map for lung cancer from GSE5364 and its degree distribution.
Figure S3: Hierarchical clustering in the M-P link map for liver cancer from GSE5364.
Figure S4: Hierarchical clustering in the M-P link map for lung cancer from GSE5364.
TABLE S1: The characteristics of molecules with the sM-mP pattern in liver cancer.
TABLE S2: The characteristics of molecules with the sM-mP pattern in lung cancer.
TABLE S3: Details of the validation analysis by comparing the relationship of small molecules targeting pathways to DrugBank and CTD databases for breast, liver and lung cancers.

## Figures and Tables

**Table 1:** The characteristics of molecules with the sM-mP pattern in breast cancer.

Small molecule	Degree in M-P link map	Description of the small molecule
Tanespimycin	16	Antineoplastic antibiotic, HSP90 inhibitor
Monorden	13	HSP90 inhibitor, DNA topoisomerase VI inhibitor
Vorinostat	12	Treats cutaneous T cell lymphoma and breast neoplasm
Adiphenine	11	Antispasmodic agent
LY-294002	10	PI3 kinase inhibitor
Alvespimycin	10	Antineoplastic antibiotic, HSP90 inhibitor
Monensin	10	Blocks intracellular protein transport and exhibits antibiotic and antimalarial efficacy
Biperiden	10	Unknown
