# Supplementary material for: Integrated Analysis Identifies Interaction Patterns between Small Molecules and Pathways
Source: Biomed Res Int. 2014 Jul 13;2014:931825. doi: 10.1155/2014/931825 (PMC4121214; doi:10.1155/2014/931825)
Supplement: Supplementary file 1 — Figure S1: Visualisation of the M-P link map for liver cancer from GSE5364 and its degree distribution. Figure S2: Visualisation of the M-P link map for lung cancer from GSE5364 and its degree distribution. Figure S3: Hierarchical clustering in the M-P link map for liver cancer from GSE5364. Figure S4: Hierarchical clustering in the M-P link map for lung cancer from GSE5364. TABLE S1: The characteristics of molecules with the sM-mP pattern in liver cancer. TABLE S2: The characteristics of molecules with the sM-mP pattern in lung cancer. TABLE S3: Details of the validation analysis by comparing the relationship of small molecules targeting pathways to DrugBank and CTD databases for breast, liver and lung cancers. [file 931825.f1.pdf]

Supplementary materials:

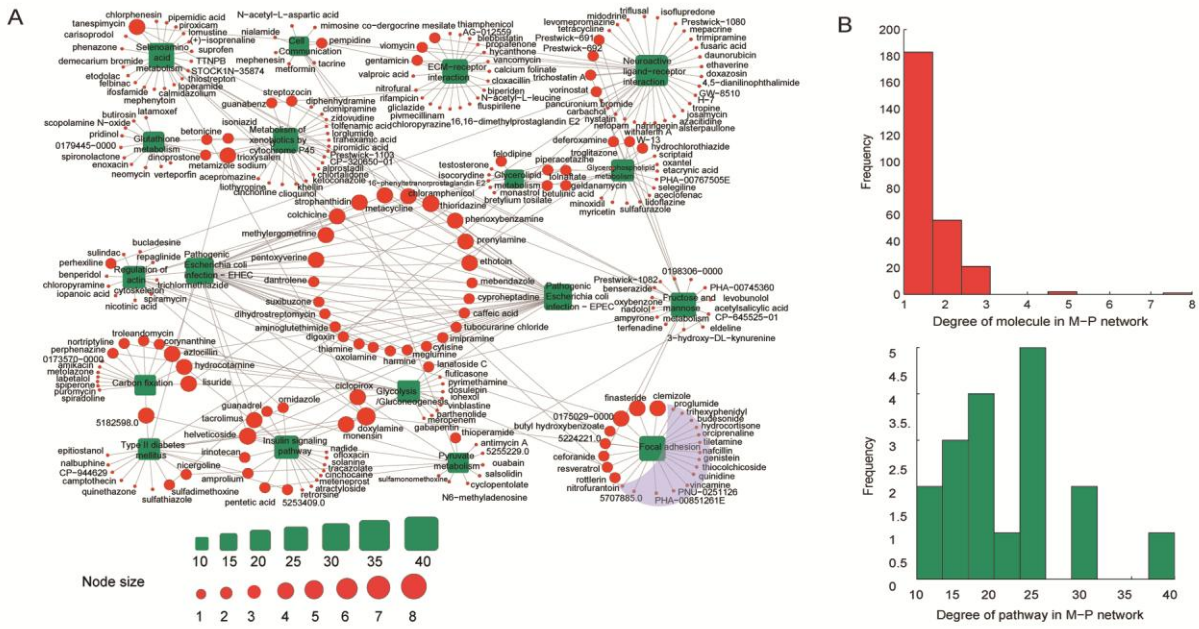

FIGURE S1: Visualisation of the M-P link map for liver cancer and its degree distribution. (A) The red circles and green rectangles correspond to the small molecules and metabolic pathways, respectively. Node size is proportional to the degree of the node. (B) The degree distribution of small molecules and pathways.

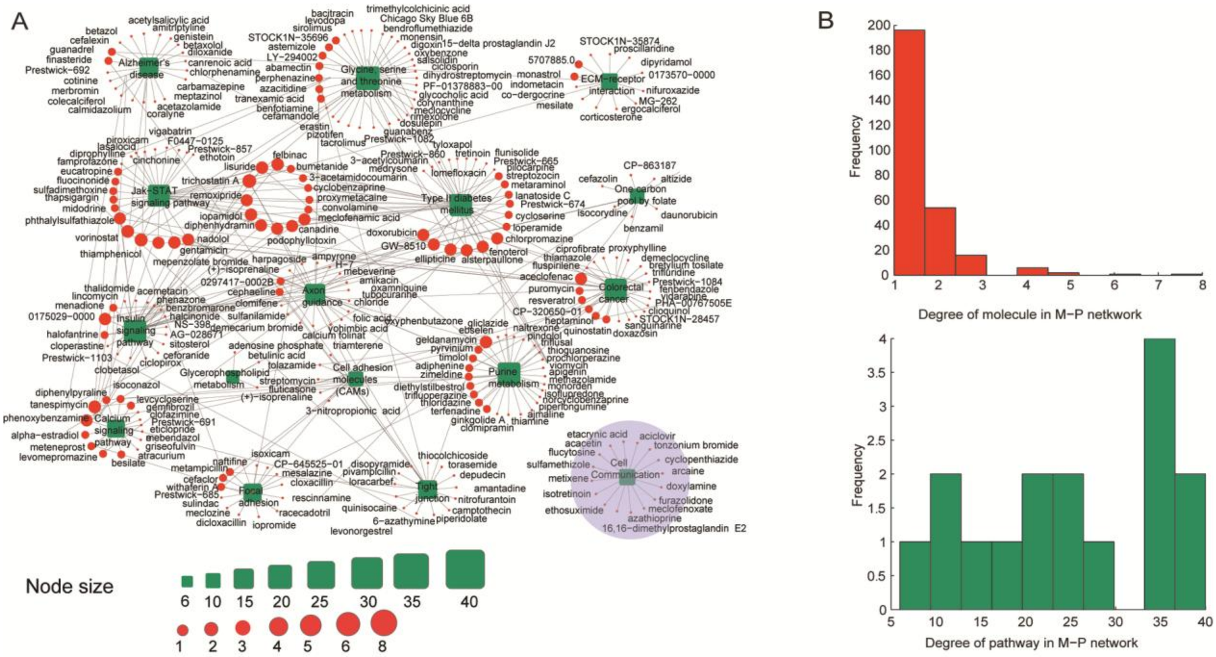

FIGURE S2: Visualisation of the M-P link map for lung cancer and its degree distribution. (A) The red circles and green rectangles correspond to the small molecules and metabolic pathways, respectively. Node size is proportional to the degree of the node. (B) The degree distribution of small molecules and pathways.

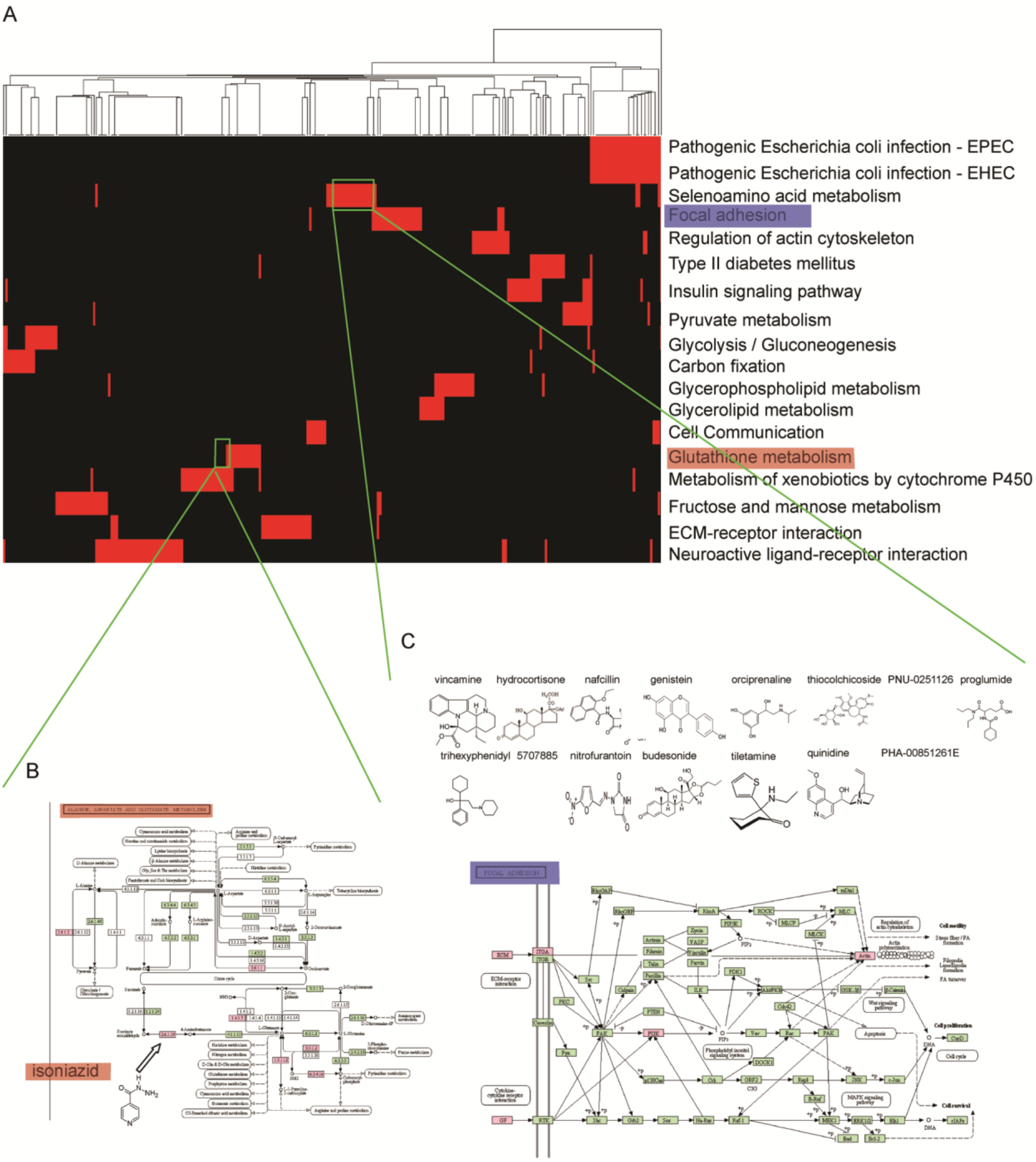

FIGURE S3. Hierarchical clustering in the M-P link map for liver cancer from GSE5364. (A) Hierarchical clustering between 263 small molecules and 18 pathways. The corresponding cells are coloured red where small molecules link to the pathways in the M-P link map. The labels for the corresponding pathways are shown on the right of the figure. (B) Zoomed-in plot of an sM-sP link between isoniazid and the glutathione metabolism pathway. The gene indicated by the arrow is the drug target of isoniazid. Differentially expressed genes in this pathway are coloured pink, while other genes in green are human disease-related genes. (C) Zoomed-in plot of mM-sP links between 15 small molecules and the focal

adhesion. These 15 small molecules target only the focal adhesion pathway. Their structures are given. The differentially expressed genes in this pathway are coloured pink, while other genes in green are human disease-related genes.

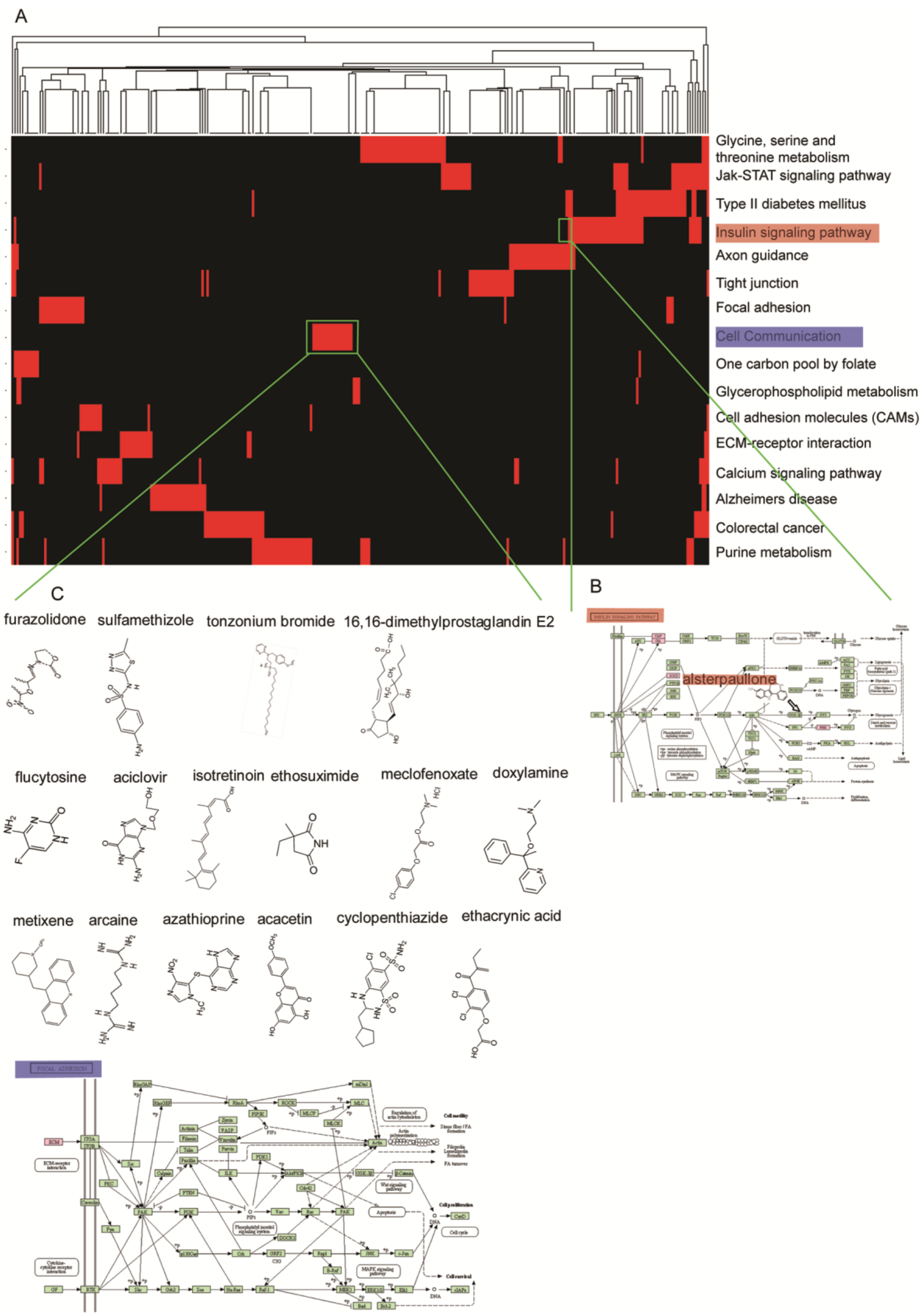

FIGURE S4: Hierarchical clustering in the M-P link map for lung cancer from GSE5364. (A) Hierarchical clustering between 276 small molecules and 16 pathways. The corresponding cells are coloured red where small molecules link to the pathways in the M-P link map. The labels for the corresponding pathways are shown on the right of the figure. (B) Zoomed-in plot of an sM-sP link between alsterpaullone and the

insulin signaling pathway. The gene indicated by the arrow is the drug target of alsterpaullone. Differentially expressed genes in this pathway are coloured pink, while other genes in green are human disease-related genes. (C) Zoomed-in plot of mM-sP links between 16 small molecules and the Cell Communication. Their structures are given. The differentially expressed genes in this pathway are coloured pink, while other genes in green are human disease-related genes.

TABLE S1:. The characteristics of molecules with the sM-mP pattern in liver cancer

Small molecule	Degree in M-P link map	Description of the small molecule
doxylamine	8	used s an antitussive, antiemetic, and hypnotic
helveticoside	5	unknown
thioridazine	5	a phenothiazine antipsychotic agent

TABLE S2: The characteristics of molecules with the sM-mP pattern in lung cancer

Small molecule	Degree in M-P link map	Description of the small molecule
trichostatin A	8	serves as an antifungal antibiotic
vorinostat	6	as histone deacetylase inhibitors
thiamphenicol	5	an antibiotic
tanespimycin	5	treatment of cancer

direct evidence f	mM_GrugE	mM_name	mM_Description	target_GeneSym
	DB00227	Lovastatin	Lovastatin is a cholesterol-lowering age	ITGAL
	DB00877	Sirolimus	Sirolimus is a potent immunosuppressa	MTOR
	DB01118	Amiodarone	It increases the duration of ventricular	ADRB1
	DB01235	L-DOPA	It is used for the treatment of parkinso	DRD1
	DB01235	L-DOPA	It is used for the treatment of parkinso	DRD2
	DB00589	Lisuride	An ergot derivative that acts as an agor	HTR2A
	DB00589	Lisuride	An ergot derivative that acts as an agor	HTR2B
	DB00589	Lisuride	An ergot derivative that acts as an agor	HTR2C
	DB04820	Nialamide	Withdrawn from the Canadian, US, and	MAOA
	DB04820	Nialamide	Withdrawn from the Canadian, US, and	MAOB
	DB01041	Thalidomide	Thalidomide displays immunosuppresiv	TNF
marker/mechani	DB01645	Genistein	It is used as an antineoplastic and antit	PTK2B
	DB08814	Triflusal	Triflusal appears to be equally effective	NFKB1
	DB00877	Sirolimus	Sirolimus is a potent immunosuppressa	MTOR
	DB00811	Ribavirin	A nucleoside antimetabolite antiviral ag	ADK
	DB00811	Ribavirin	A nucleoside antimetabolite antiviral ag	ENPP1
	DB00811	Ribavirin	A nucleoside antimetabolite antiviral ag	IMPDH1
	DB00811	Ribavirin	A nucleoside antimetabolite antiviral ag	NT5C2
	DB01037	Selegiline	It is used in newly diagnosed patients w	MAOA
	DB01037	Selegiline	It is used in newly diagnosed patients w	MAOB
	DB04841	Flunarizine	It is effective in the prophylaxis of migr	CACNA1G
	DB02375	Myricetin	Not Available	PIK3CG
	DB01055	Mimosine	An antineoplastic alanine-substituted p	TYR
	DB03585	Oxyphenbutazo	Oxyphenbutazone was withdrawn from	PLA2G2E

KEGG_pathway	Pathway_Description
Leukocyte transendothelial migratio	Leukocyte migaration from the blood into tissu
Adipocytokine signaling pathway	Increased adipocyte volume and number are p
Gap junction	Gap junctions contain intercellular channels th
Gap junction	Gap junctions contain intercellular channels th
Gap junction	Gap junctions contain intercellular channels th
Gap junction	Gap junctions contain intercellular channels th
Gap junction	Gap junctions contain intercellular channels th
Gap junction	Gap junctions contain intercellular channels th
Glycine, serine and threonine metal	Serine is derived from 3-phospho-D-glycerate,
Glycine, serine and threonine metal	Serine is derived from 3-phospho-D-glycerate,
Hematopoietic cell lineage	Blood-cell development progresses from a her
Leukocyte transendothelial migratio	Leukocyte migaration from the blood into tissu
MAPK signaling pathway	The mitogen-activated protein kinase (MAPK)
mTOR signaling pathway	
Purine metabolism	
Purine metabolism	
Purine metabolism	
Purine metabolism	
Tryptophan metabolism	
Tryptophan metabolism	
Type II diabetes mellitus	"Diabetogenic" factors including FFA, TNFalpha
Type II diabetes mellitus	"Diabetogenic" factors including FFA, TNFalpha
Tyrosine metabolism	
VEGF signaling pathway	There is now much evidence that VEGFR-2 is tl

ues is vital for immune surveillance and inflammation. During this diapedesis of leukocytes, the leukocyte
ositively correlated with leptin production, and negatively correlated with production of adiponectin.
at allow direct communication between the cytosolic compartments of adjacent cells. Each gap junction
at allow direct communication between the cytosolic compartments of adjacent cells. Each gap junction
at allow direct communication between the cytosolic compartments of adjacent cells. Each gap junction
at allow direct communication between the cytosolic compartments of adjacent cells. Each gap junction
at allow direct communication between the cytosolic compartments of adjacent cells. Each gap junction
at allow direct communication between the cytosolic compartments of adjacent cells. Each gap junction
an intermediate of glycolysis, and glycine is derived from serine. Threonine is an essential amino acid, w
an intermediate of glycolysis, and glycine is derived from serine. Threonine is an essential amino acid, w
natopoietic stem cell (HSC), which can undergo either self-renewal or differentiation into a multilineage
ues is vital for immune surveillance and inflammation. During this diapedesis of leukocytes, the leukocyte
cascade is a highly conserved module that is involved in various cellular functions, including cell prolifera

a and cellular stress induce insulin resistance through inhibition of IRS1 functions. Serine/threonine phos
a and cellular stress induce insulin resistance through inhibition of IRS1 functions. Serine/threonine phos

he major mediator of VEGF-driven responses in endothelial cells and it is considered to be a crucial signal

es bind to endothelial cell adhesion molecules (CAM) and then migrate across the vascular endothelium.

channel is formed by docking of two 'hemichannels', each containing six connexins, contributed by eac
channel is formed by docking of two 'hemichannels', each containing six connexins, contributed by eac
channel is formed by docking of two 'hemichannels', each containing six connexins, contributed by eac
channel is formed by docking of two 'hemichannels', each containing six connexins, contributed by eac
channel is formed by docking of two 'hemichannels', each containing six connexins, contributed by eac
channel is formed by docking of two 'hemichannels', each containing six connexins, contributed by eac
hich animals cannot synthesize. In bacteria and plants, threonine is derived from aspartate.
hich animals cannot synthesize. In bacteria and plants, threonine is derived from aspartate.
committed progenitor cell: a common lymphoid progenitor (CLP) or a common myeloid progenitor (CM
es bind to endothelial cell adhesion molecules (CAM) and then migrate across the vascular endothelium.
tion, differentiation and migration.

phorylation, interaction with SOCS, regulation of the expression, modification of the cellular localization
phorylation, interaction with SOCS, regulation of the expression, modification of the cellular localization

l transducer in both physiologic and pathologic angiogenesis.

neighboring cell. These channels permit the direct transfer of small molecules including ions, amino ac
neighboring cell. These channels permit the direct transfer of small molecules including ions, amino ac
neighboring cell. These channels permit the direct transfer of small molecules including ions, amino ac
neighboring cell. These channels permit the direct transfer of small molecules including ions, amino ac
neighboring cell. These channels permit the direct transfer of small molecules including ions, amino ac
neighboring cell. These channels permit the direct transfer of small molecules including ions, amino ac

P).

, and degradation represent the molecular mechanisms stimulated by them.
, and degradation represent the molecular mechanisms stimulated by them.

ids, nucleotides, second messengers and other metabolites between adjacent cells.
ids, nucleotides, second messengers and other metabolites between adjacent cells.
ids, nucleotides, second messengers and other metabolites between adjacent cells.
ids, nucleotides, second messengers and other metabolites between adjacent cells.
ids, nucleotides, second messengers and other metabolites between adjacent cells.
ids, nucleotides, second messengers and other metabolites between adjacent cells.
